# Supplementary material for: Altered epithelial development of the lateral ventricle choroid plexus in Mllt11 mutants
Source: Biol Open. 2025 Jul 21;14(7):bio061966. doi: 10.1242/bio.061966 (PMC12320971; doi:10.1242/bio.061966)
Supplement: Supplementary file 1 [file biolopen-14-061966_review_history.pdf]

Biology Open

Altered epithelial development of the lateral ventricle choroid plexus in Mllt11 mutants

Sam Moore, Danielle Stanton-Turcotte, Emily A. Witt and Angelo Iulianella
DOI: 10.1242/bio.061966

Editor: Tristan Rodr-guez

Review timeline
Original submission: 27 February 2025
Editorial decision: 13 March 2025
First revision received: 17 June 2025
Accepted: 18 June 2025

Original submission

First decision letter

MS ID#: bio.061966

MS Title: Altered epithelial development of the lateral ventricle choroid plexus in Mllt11 mutants

Authors: Sam Moore, Danielle Stanton-Turcotte, Emily A. Witt and Angelo Iulianella

I have now reached a decision on the above manuscript.

The reviewer reports are shown at the bottom of this email or can be accessed, together with a copy of this decision letter, by going to:

As you will see, the reviewers raised a number of substantial criticisms that prevent me from accepting the paper at this stage.

They suggest, however, that a revised version might prove acceptable, if you can address their concerns. If you think that you can deal satisfactorily with the criticisms on revision, I would be pleased to see a revised manuscript. We would then return it to the reviewers.

At this stage, we also ask you to ensure your manuscript complies with our formatting guidelines. Provided you are able to fully address the referees' comments, we are positive about publication of your paper (we accept over 95% of revision submissions) and therefore hope you won't mind any extra work involved in reformatting your manuscript at this point.

Please ensure that you clearly highlight all changes made in the revised manuscript. Please avoid using 'Tracked changes' in Word files as these are lost in PDF conversion.

I should be grateful if you would also provide a point-by-point response detailing how you have dealt with the points raised by the reviewers in the 'Response to Reviewers' box. Please attend to all of the reviewers' comments. If you do not agree with any of their criticisms or suggestions please explain clearly why this is so.

Reviewer 1

Comments for the author

© 2025. Published by The Company of Biologists under the terms of the Creative Commons Attribution License (https://creativecommons.org/licenses/by/4.0/).

In this work, Moore and colleagues analyze the phenotypic effect of the loss of Mlltl1 in the choroid plexus (ChP) of the developing mouse brain. The study builds on previous observations of the authors of the Mlltl1 knockout, and provides an interesting and detailed characterization of the mutant ChP, that hints to possible roles in progenitor migration and epithelial cell organization.

The data is presented in a clear and concise way, is correctly analyzed and the conclusions reached by the author are based on their observations. As such, this is a valuable work.

Some minor points that may be worth addressing are the following:

1. The initial analysis of the phenotype is carried out at E14.5 (Figs 1, 2). Is this the first time point when there is an observable phenotype? How early have the authors checked for defects or changes in marker expression? This might not be required here, but it might be good to know in the case of follow up studies looking at the molecular details of the process.

2. It would be useful to complement Fig. 1 with a diagram of the anatomical regions studied at E14.5 and E18.5, indicating patterns of the Cux2-Cre driver and the markers used (LHX2, OTX1/2).

3. In Fig. 2A, B the three sections shown for each individual examined (I, II, III) should be explained in the text and/or legend.

4. What are the Ns for WTs in Fig. 2D, and for WTs and cKOs in Fig. 2E? they should be indicated in the legend.

5. Pulse labeling with EdU is an elegant assay, but the total number of doble positive OTX1/2-EdU cells per ChP appear to be slightly low (only 10-15 cells). Is this the proliferation expected to occur in 4 days of development of the ChP (from E14.5 to E18.5)? Other areas that can be seen in the sections appear to have a much higher amount of EdU+ cells. This should be discussed.

6. The layout of Fig. 3 could be improved by placing the OTX1/2-EdU immunos on top, as panels E-H, and the quantifications below as panels H, I. In this way, the text description would follow the same order as the figure.

7. The authors use a popular OTX1/2 antibody, and state it follows OTX2 expression specifically. For those not in the field, it would be useful to explain (and reference) that OTX1 is not present in these territories.

8. The authors should define "NEP" the first time they use it (in the abstract).

9. When mentioning genes, italics should be used, and when referring to protein, all caps (for example, OTX1/2 antibody).

Reviewer 2

Comments for the author

Moore et al. investigate the role of the transcription factor Mllt11 in forming the choroid plexus. They suggest that defects exist in the migration of cells into a part of the choroid plexus. While the study is potentially interesting, clarification is needed.

One of the issues I have is the use of pseudostratified epithelium. I am not sure if the ChP stalk is an example of pseudostratified epithelium (i.e., an epithelium where every cell connects with the basal lamina and has an apical side that contacts a ventricle, with nuclei at different levels depending on the cell cycle). This is detailed here https://journals.biologists.com/jcs/article/130/11/1859/56156/Pseudostratified-epithelia-cell-biology-diversity .WT epithelia appear columnar and the nuclei are all at similar apicobasal positions. Mutants seem to exhibit some degree of stratification (rather than pseudostratification)

© 2025. Published by The Company of Biologists under the terms of the Creative Commons Attribution License (https://creativecommons.org/licenses/by/4.0/).

in their epithelia. It would be useful to investigate laminin/fibronectin staining in these mutants, as that would provide a better idea of epithelial organisation.

The increase in Lhx2+ cells and a decrease in Otx1/2 positive cells suggest a shift in the Lhx2 cKO phenotype (where CH is expanded at the expense of the SVZ). Would the authors consider this a migration defect? Or does this indicate a failure in lineage selection? Is there precedent for the involvement of the Wnt signalling pathway in Mllt11 function? Some of the extensive work that has been done on Lhx2 should be cited, and these ideas can be explored further. Is is possible that the CH and ChP relationship is also based in lineage selection and not on migration?

WT controls are the Cre-line driving TdTomato. It would be useful to use the non-Cre expressing littermate control of the Cux2-Cre:Ai9:Mllt11flox/flox to determine any background effects on ChP size. This is a more appropriate control. The number of experimental samples is stated as 3-6. It would be beneficial to provide actual numbers for each experiment. Data has been subjected to appropriate statistical tests.

Reviewer's Responses to Questions

Experimental quality
Does each figure have the proper controls?
If 'No', please indicate reasons in Comments for Author box below.

Reviewer #1:

- Yes

Reviewer #2:

- No

Were the data analyzed using appropriate statistical tests?
If 'No', please indicate reasons in Comments for Author box below.

Reviewer #1:

- Yes

Reviewer #2:

- Yes

Reproducibility
Were experiments performed using adequate number of biological replicates?
If 'No', please indicate reasons in Comments for Author box below.

Reviewer #1:

- Yes

Reviewer #2:

- No

© 2025. Published by The Company of Biologists under the terms of the Creative Commons Attribution License (https://creativecommons.org/licenses/by/4.0/).

Does the methods section provide sufficient detail to permit reproducibility?
If 'No', please indicate reasons in Comments for Author box below.

Reviewer #1:

- Yes

Reviewer #2:

- Yes

Completeness
Are the manuscript's conclusions supported by the data?
If 'No', please indicate reasons in Comments for Author box below.

Reviewer #1:

- Yes

Reviewer #2:

- No

Scholarship
Do the authors cite and discuss the merits of data that would argue for and against their conclusion?
If 'No', please indicate reasons in Comments for Author box below.

Reviewer #1:

- Yes

Reviewer #2:

- No

Does the manuscript title & abstract accurately reflect the contents of the manuscript, without hyperbole?
If 'No', please indicate reasons in Comments for Author box below.

Reviewer #1:

- Yes

Reviewer #2:

- No

© 2025. Published by The Company of Biologists under the terms of the Creative Commons Attribution License (https://creativecommons.org/licenses/by/4.0/).

First revision

Author response to reviewers' comments

Dear Dr. Tristan A. Rodriguez.
We thank reviewers for their insightful comments and suggestions to improve our short report study on the role of the novel protein Mllt11 in the formation of mammalian lateral ventricle choroid plexus (ChP). We now submit our revised manuscript which addresses the major concerns raised by the reviewers. Changes are highlighted in yellow and include an additional figure—Figure 5.
The objective of our study was to determine what was the cause of the altered choroid plexus morphology in Mllt11 mouse mutants. Using *Cux2* $^{iresCre/+}$ fate mapping, EdU birth dating mad epithelial cell marker analysis we initially concluded that the epithelial organization was altered to that resembling a more pseudostratified epithelial appeared, primarily based finding of ectopic epithelial cell contacts revealed by ZO-1 and Pals1 staining. However, Reviewer#2 questioned this conclusion and suggested we also look at basal lamina markers to probe more deeply into the epithelial tissue relationships. In our revised manuscript, we incorporated this reviewers' suggestions and added a new figure (Figure 5), which depicts Laminin staining of the basal lamina of the neonatal ChP epithelia. We also revised Figure 1 to include a summary diagram, and Figure 3, according to the suggestions of reviewer 1. We noted no differences between Mllt11 conditional knockout (cKO) mutants and wild type controls. We therefore tempered our conclusions about the change to pseudostratified epithelial but maintained that there was a thickening of the epithelia due to an abnormal juxtapositioning of epithelial cells. This likely contributed to the shortened ChP stalks in the Mllt11 mutants. We modified the title of our manuscript, which now is: *"Altered epithelial development of the lateral ventricle choroid plexus in Mllt11 mutants"*. We added a new co-author to the study, Emily A. Witt, who did the Laminin staining experiments and quantifications.
We maintain that the origin of the *Mllt11* mutant ChP phenotype is likely due to altered ChP progenitor migration and epithelial thickening, leading to shorter ChP at birth. This is a novel finding and sheds light on the molecular regulation of ChP development. We hope that our study now meets the standard for acceptance to *Biology Open*.
We address the reviewers' comments point-by-point below.

REVIEWER 1:

In this work, Moore and colleagues analyze the phenotypic effect of the loss of Mlltl1 in the choroid plexus (ChP) of the developing mouse brain. The study builds on previous observations of the authors of the Mlltl1 knockout, and provides an interesting and detailed characterization of the mutant ChP, that hints to possible roles in progenitor migration and epithelial cell organization.

The data is presented in a clear and concise way, is correctly analyzed and the conclusions reached by the author are based on their observations. As such, this is a valuable work.

Some minor points that may be worth addressing are the following:
1. The initial analysis of the phenotype is carried out at E14.5 (Figs 1, 2). Is this the first time point when there is an observable phenotype? How early have the authors checked for defects or changes in marker expression? This might not be required here, but it might be good to know in the case of follow up studies looking at the molecular details of the process.

Response: We thank the reviewer for their comments. We evaluated most markers at E18.5 as that is when the choroid plexus (ChP) phenotype is most obvious. For fate mapping studies and cortical hem patterning, we examined phenotypes at E14.5, when hem patterning has occurred. We agree with the reviewer that more molecular details would be useful in follow up studies. The goal of the current study was to identify the cellular origin of the shortened ChP stalk phenotype in Mllt11 mutants, and we believe our analysis has been successful in this regard.

2. It would be useful to complement Fig. 1 with a diagram of the anatomical regions studied at E14.5 and E18.5, indicating patterns of the Cux2-Cre driver and the markers used (LHX2, OTX1/2).

Response: We thank the reviewer for this suggestion and have now added a summary diagram in figure 1 (**Fig. 1I**) that maps out Cux2-Cre activity and Lhx2, Otx1, Otx2 expression. In addition, we previously wrote a review (Moore and Iulianella, 2021, *Open Biol* 11, 210042) on cortical hem patterning that we referenced in our manuscript.

3. In Fig. 2A, B the three sections shown for each individual examined (I, II, III) should be explained in the text and/or legend.

© 2025. Published by The Company of Biologists under the terms of the Creative Commons Attribution License (https://creativecommons.org/licenses/by/4.0/).

Response: We have added an explanation to Figure 2 legend.

4. What are the Ns for WTs in Fig. 2D, and for WTs and cKOs in Fig. 2E? they should be indicated in the legend.

Response: We have added the n's (N=9) for WTs and cKOs to the text and the legend of Figure 2.

5. Pulse labeling with EdU is an elegant assay, but the total number of doble positive OTX1/2-EdU cells per ChP appear to be slightly low (only 10-15 cells). Is this the proliferation expected to occur in 4 days of development of the ChP (from E14.5 to E18.5)? Other areas that can be seen in the sections appear to have a much higher amount of EdU+ cells. This should be discussed.

Response: The choroid plexus first appears at E11 and grows rapidly into a large recognizable structure by E14. (Sturrock 1979 *J.Anat* **129**, 777-793). Maturation of the Choroid Plexus continues up through the first postnatal weeks (Fame and Lehtinen, 2020, Dev Cell, **52**, 261-275). In order to capture Otx1/2$^+$ progenitors we administered a single pulse at E14.5 when choroid growth is occurring and analysed animals at E185. A single dose of EdU was administered to prevent resorption of litters which in our hands can occur with repeated daily dosing of EdU. While this approach labelling only a few Otx1/2$^+$/EdU$^+$, it provided a clear cellular resolution of the treadmilling behavior of NEPs entering the from dorsal to ventral halves of the choroid.

6. The layout of Fig. 3 could be improved by placing the OTX1/2-EdU immunos on top, as panels E-H, and the quantifications below as panels H, I. In this way, the text description would follow the same order as the figure.

Response: We have altered the layout of figure 3 as described and edited the figure legend and text to reflect the changes

7. The authors use a popular OTX1/2 antibody, and state it follows OTX2 expression specifically. For those not in the field, it would be useful to explain (and reference) that OTX1 is not present in these territories.

Response: We thank the reviewer for this suggestion and have now added a description of Otx1 and Otx2 expression patterns to the text as well as a diagram of the expression patterns in the revised Fig. 1I.

8. The authors should define "NEP" the first time they use it (in the abstract).

Response: We have now included a definition of "NEP" in the abstract.

9. When mentioning genes, italics should be used, and when referring to protein, all caps (for example, OTX1/2 antibody).

Response: The mouse nomenclature indicated the first letter of the protein is capitalized, whereas human nomenclature is all caps. We opted to maintain the first letter capitalized when referring to protein, and italics when referring to the gene or mRNA.

REVIEWER 2:

Moore et al. investigate the role of the transcription factor Mllt11 in forming the choroid plexus. They suggest that defects exist in the migration of cells into a part of the choroid plexus. While the study is potentially interesting, clarification is needed.

1. One of the issues I have is the use of pseudostratified epithelium. I am not sure if the ChP stalk is an example of pseudostratified epithelium (i.e., an epithelium where every cell connects with the basal lamina and has an apical side that contacts a ventricle, with nuclei at different levels depending on the cell cycle). This is detailed here https://journals.biologists.com/jcs/article/130/11/1859/56156/Pseudostratified-epithelia-cell-biology-diversity .WT epithelia appear columnar and the nuclei are all at similar apicobasal positions. Mutants seem to exhibit some degree of stratification (rather than pseudostratification) in their epithelia. It would be useful to investigate laminin/fibronectin staining in these mutants, as that would provide a better idea of epithelial organisation.

Response: We thank the reviewer for the constructive criticism and for reconsideration of the epithelial phenotypes we observed. We agree that the ChP is not an example of a pseudostratified epithelium and include a section in the discussion of the type of epithelial morphologies exhibited by the choroid during its maturation. We have followed the reviewer's

© 2025. Published by The Company of Biologists under the terms of the Creative Commons Attribution License (https://creativecommons.org/licenses/by/4.0/).

suggestion and now include a new figure (**Fig. 5**) that contains an analysis of lamina staining in the control vs *Mllt11* cKO lateral ventricle ChP at E18.5. We observed no significant changes in Laminin distribution following *Mllt11* loss. The *Mllt1*1 cKO mutant phenotype displays aberrantly displaced epithelial cells that nonetheless maintains, as far as we can tell, the appropriate apical (identified by Pals1 staining) and basal lamina (identified by Laminin staining) boundaries. We speculate that the aberrant epithelial cell layer can either reflect a migration defects or epithelial transition defect. In light of tehse new findinsg we have removed our language on pseudostratified transformation, tempered our conclusions and study title , which now reads: "*Altered epithelial development of the lateral ventricle choroid plexus in Mllt11 mutants*".

2. The increase in Lhx2+ cells and a decrease in Otx1/2 positive cells suggest a shift in the Lhx2 cKO phenotype (where CH is expanded at the expense of the SVZ). Would the authors consider this a migration defect? Or does this indicate a failure in lineage selection? Is there precedent for the involvement of the Wnt signalling pathway in Mllt11 function? Some of the extensive work that has been done on Lhx2 should be cited, and these ideas can be explored further. Is is possible that the CH and ChP relationship is also based in lineage selection and not on migration?

Response: The reviewer raises an interesting point, however, it is unclear if the increase in Lhx2+ cells in the *Mllt11* cKO represents a lineage fate shift or migration defect. In cancer cell lines, Mllt11 (also known as Tcf7co-factor) appears to modulate canonical Wnt signaling, which would tie in nicely with the Lhx2+ phenotype. However, as a cautionary note, there is no evidence yet in embryos or untransformed cell lines of a Wnt-Mllt11 connection. Mllt11 is an enigmatic protein with no identifiable interaction motifs except for a nuclear export sequence. In a previous study (Stanton-Turcotte et al, 2022, *J Neursci* **42**, 3931-3948), we used fetal brain lysates for proteomics and identify several cytoskeletal proteins. This is why we are reluctant to discuss possible transcriptional roles for Mllt11 in the study, as cancer cell lines can reflect aberrant regulatory processes with little relevance to normal development. We are exploring the Mllt11-Wnt connection in cultured neural progenitor cell lines, but as the work is ongoing it lies outside the purview of the current study, which tightly focused on the cellular origin of the ChP phenotype as a short report. We inserted the following sentence in the "Conclusion" section of our manuscript: *"Interestingly, the reduced CH region was associated with an expanded Lhx2$^+$ DNE region in the cKOs, suggesting that Mllt11 may regulate CH vs DNE fate choice; the molecular mechanism of this regulation is currently unknown."*

Alternatively, we did note a significant reduction of tomato$^+$ Cux2-Cre fate cells near the base of the ChP (Fig. 1G, H). In our view this likely reflects a migration defect contributing to the shortened ChP stalk phenotype in *Mllt11* mutants, which we discussed in our study.

3. WT controls are the Cre-line driving TdTomato. It would be useful to use the non-Cre expressing littermate control of the Cux2-Cre:Ai9:Mllt11flox/flox to determine any background effects on ChP size. This is a more appropriate control.

Response: We respectfully disagree with the reviewer and support the developing consensus opinion that Cre expressing controls are the appropriate control in mouse transgenic studies. Cre Recombinase, unfortunately, is known to produce phenotypes all on its own. For this reason, it is highly recommended in the literature that Cre expressing controls be used (e.g. Song and Palmiter, 2018, *Trends Genet*, **34**, 333-340; Giusti et al, 2014, *J Psychiatr Res* **55**, 87-95; Bhandary and Robbins, 2015, *J Mol Cell Cardiol*, **86**, 136-137). This is why we have long since used Cre heterozygotes as appropriate controls for our studies using genetically engineered mice.

4. The number of experimental samples is stated as 3-6. It would be beneficial to provide actual numbers for each experiment. Data has been subjected to appropriate statistical tests.

Response: We have updated the text to include the actual sample numbers for each experiment. It varies from n= 3 to 9 for each genotype depending on the experiment, which we clarified in the methods and combined results/discussion sections as well as the figure legends.

Second decision letter

MS ID#: bio.061966R1

© 2025. Published by The Company of Biologists under the terms of the Creative Commons Attribution License (https://creativecommons.org/licenses/by/4.0/).

MS Title: Altered epithelial development of the lateral ventricle choroid plexus in Mllt11 mutants

Authors: Sam Moore, Danielle Stanton-Turcotte, Emily A. Witt and Angelo Iulianella

I am happy to tell you that your manuscript has been accepted for publication in Biology Open, pending our standard publication integrity checks. It was accepted on 18 Jun 2025.

© 2025. Published by The Company of Biologists under the terms of the Creative Commons Attribution License (https://creativecommons.org/licenses/by/4.0/).
